# Exogenous Ergothioneine and Glutathione Limit Postharvest Senescence of Arugula

**DOI:** 10.3390/antiox13091140

**Published:** 2024-09-21

**Authors:** Dhanya Sivakumar, Gale Bozzo

**Affiliations:** Department of Plant Agriculture, University of Guelph, Guelph, ON N1G 2W1, Canada; sivakumd@uoguelph.ca

**Keywords:** arugula, ascorbate, ergothioneine, glutathione, postharvest, senescence

## Abstract

Arugula is susceptible to postharvest deterioration. We tested the impact of exogenous antioxidant (i.e., ergothioneine and glutathione) dip solutions on arugula quality during storage at 4 °C or 10 °C for up to 17 days relative to a non-antioxidant treatment. Leaves from each dip treatment and storage temperature were assessed for visual quality and endogenous antioxidant metabolite profiles. Overall, leaf discolouration, wilting, and decay were more rapid at 10 °C than at 4 °C. Both antioxidant treatments limited leaf discolouration at 4 °C. Exogenous ergothioneine reduced wilting at 4 °C, whereas exogenous glutathione limited the incidence of leaf decay. At 10 °C, glutathione reduced the incidence of discolouration and decay, whereas both antioxidant dip treatments limited the decline in leaf yellowing. Ergothioneine was solely detected in ergothioneine-treated leaves; a decrease occurred within the first two days of storage but was unchanged thereafter. Although both antioxidant treatments did not affect endogenous glutathione concentrations at either storage temperature, glutathione disulfide was stable within the glutathione-treated leaves, whereas it increased in the other treatments. Ascorbate degradation was delayed in ergothioneine-treated leaves at 4 °C relative to all other treatments, whereas both antioxidant treatments little affected ascorbate metabolism in leaves stored at 10 °C.

## 1. Introduction

*Diplotaxis tenuifolia* L. (DC.) and its related species *Eruca sativa* (Mill.) are members of the *Brassicaceae* family. Both are commonly known as arugula or rocket. Arugula is known for its sharp flavour characteristics. Arugula leaves contain glucosinolates and flavonols, some of which are proposed as beneficial for human health [1]. For the retail sector, arugula is marketed at various stages of leaf maturity. Typically, baby leaf vegetables, including arugula, are harvested in advance of full maturity and not beyond the eight true-leaf stage of the plant [2]. Arugula can be grown indoors under controlled environments, which affords producers and consumers a year-round supply [3]. 

The optimal conditions for postharvest storage of arugula are temperatures in the range of 0 to 2 °C and relative humidities ≥ 95% [4]. Baby leaf arugula has a shelf life of 16 days when stored at temperatures ranging from 0 to 3 °C [5,6]. Unfortunately, there is evidence that many vegetables can be exposed to a wide range of temperatures (i.e., −1 to 19 °C) within refrigerated display cabinets that are used in the retail sector [7]. Many leafy green *Brassicaceae* vegetables tend to be highly perishable in the retail sector [8]. The shelf life of fresh arugula is reduced with temperature abuse (i.e., warmer temperatures than those recommended for optimal storage) [5,6,9]. Arugula shelf life terminates with the senescence-related loss of green colour and appearance of yellowing, as well as wilting and spoilage due to microbial infection [6,10,11]. Senescence is accelerated with storage under temperature abuse (e.g., ≥5 °C) [12,13]. The development of off-flavours can also occur with postharvest storage of wild and cultivated arugula leaves [13,14,15]. 

Postharvest senescence of horticultural commodities is coupled to alterations in cellular redox. This includes a rise in reactive oxygen species (ROS) concentrations, which can elicit negative effects such as lipid peroxidation and damage to cellular components such as DNA, proteins, and membranes that culminate in senescence-related signs of deterioration [16]. Plants utilise various enzymatic and non-enzymatic mechanisms to convert ROS (e.g., H_2_O_2_) into less reactive compounds (e.g., water). The Foyer–Halliwell–Asada pathway is the primary mechanism for ROS detoxification in plants. This pathway is dependent upon the cellular availability of the antioxidants glutathione (GSH) and ascorbate [17]. The Foyer–Halliwell–Asada pathway includes ascorbate peroxidase, which catalyses the conversion of H_2_O_2_ to water in the presence of ascorbate, an electron donor. The ascorbate peroxidase reaction yields monodehydroascorbate, which is converted to dehydroascorbate (DHA). Ascorbate is regenerated from DHA in the presence of DHA reductase (DHAR), a GSH-dependent enzyme that yields glutathione disulfide (GSSG) as a secondary product. The ratio of GSH/GSSG (i.e., glutathione redox status) is ≥20:1 in non-stressed plants [18]. A signature response of oxidative stress in postharvest senescence is a decrease in GSH/GSSG and/or ascorbate/DHA ratios [17]. DHA and GSSG can accumulate with postharvest senescence in the absence of these recycling steps. For example, DHA and GSSG accumulate in Chinese flowering cabbage and pak choi during storage at 15 °C and 20 °C, respectively [19,20]. The oxidation of ascorbate to DHA tends to be more pronounced and rapid at suboptimal temperatures than at the low storage temperatures recommended for vegetables, including arugula. For example, a near 50% loss of ascorbate was evident in arugula after 3 days at 15 °C, whereas less than a 30% loss of this antioxidant occurred after 10 days at 0 °C [13]. Larger ascorbate losses have been described for chilled lettuce [21]. Moreover, baby arugula leaves tend to undergo more ascorbate degradation than mature leaves [6]. 

The application of exogenous GSH delays the postharvest deterioration of some horticultural crops. Specifically, chilling injury of bell peppers is limited when exogenous GSH is applied prior to storage [22]. This phenomenon is associated with greater ascorbate and GSH concentrations but less H_2_O_2_ relative to untreated fruit. Less browning and weight loss occurred in okra that was dipped into a GSH solution prior to storage at 10 °C [23]. Similarly, Ali et al. [24] found that less storage-related browning developed in lotus root slices treated with exogenous GSH as compared to antioxidant-free root slices. Both the okra and lotus studies established that the control of browning by GSH is associated with increased ascorbate redox status.

Ergothioneine (EGT) is a potent antioxidant that is more efficient than GSH at detoxifying singlet oxygen and the hydroxyl radical [25]. Although GSH occurs in many organisms, including plants, EGT is biosynthesised in fungi and some bacteria. This includes culinary mushrooms (e.g., *Agaricus bisporus*, *Pleurotus ostreatus*), certain yeasts (e.g., *Schizosaccharomyces pombe*) and the bacterium *Lactobacillus casei* [26,27]. Melanosis is inhibited in shrimp [28] and crab [29] following treatment with EGT or mushroom extracts containing EGT. Moreover, exogenous EGT limits the discolouration of ground beef and minced tuna [30]. EGT reduces the bacterial spoilage of Japanese sea bass [31]. Finally, Qian et al. [32] demonstrated exogenous EGT limits postharvest browning of white button mushrooms; this treatment also stabilised the ascorbate concentration within the stored mushrooms. Nevertheless, there is no information on the impact of exogenous EGT on any horticultural commodity. Moreover, there is also no knowledge of the impact of EGT and GSH on the postharvest quality of arugula. To the best of our knowledge, these antioxidant treatments have never been simultaneously tested on any food commodity. In this study, we investigated the effect of pre-storage EGT and GSH dip treatments on the visual quality and antioxidant profiles of baby arugula stored under the typical retail temperature of 4 °C and the suboptimal temperature of 10 °C. 

## 2. Materials and Methods

### 2.1. Plant Material, Postharvest Antioxidant Dip Treatments, and Storage

For each of three postharvest experiments, 3 kg of freshly harvested baby leaf arugula (*D. tenuifolia* ‘Letizia’; herein referred to as arugula) were acquired from a local grower. The collections were performed on the following dates in 2023: 13 March, 12 April, and 2 May. For each experiment, the harvested leaf material was immediately transported to the postharvest storage facility in the Edmund Bovey Building at the University of Guelph. 

We tested the impact of antioxidant dip treatments on the overall quality and antioxidant metabolite profiles of arugula stored at 4 °C or at 10 °C for up to 17 days. Preliminary experiments were performed where arugula was immersed in either 100 μM or 500 μM of each antioxidant treatment. Based on the smallest concentration needed to preserve the visual quality of arugula, 100 μM EGT and 500 μM GSH were selected for the in-depth postharvest experimentation as described below. A third treatment included an antioxidant-free control, where leaves were dipped into a container of Milli-Q water. All dip treatments and leaf drying thereafter were performed at ambient temperature (between 22 and 24 °C). For each treatment container, 450 g of leaves were submerged in the antioxidant or control solution under darkness. The leaves were gently inverted within the dip solution at the midway point and the end of the 30 min incubation period. Thereafter, the leaves were spun dry with salad spinners. For each treatment replicate, the leaves were partitioned into four separate non-perforated bio-based polyethylene terephthalate clamshell containers (approximate volume of 2.22 L; Good Natured Products, Vancouver, BC, Canada) and transferred into various positions within a postharvest storage room. Clamshells containing one treatment replicate were transferred to a 4 °C storage room, whereas the other treatment replicate was transferred to a second storage room set to 10 °C to simulate optimal and suboptimal retail temperatures, respectively. All storage chambers were kept under darkness. For each storage temperature/antioxidant treatment within an experiment, 50 g of leaves were randomly pooled from all corresponding clamshell containers on days 0, 2, 4, 7, and 10. Leaves held at 4 °C were also sampled on days 14 and 17. It is worth noting that on day 0, leaves were sampled 2 h after their transfer to the storage room. For each 50 g sample, a 25 g subsample was evaluated for quality indices as described below, whereas the remaining leaf material was quick frozen in liquid N_2_ and transferred to a −80 °C freezer and held under these conditions until required for metabolite analyses. 

### 2.2. Leaf Quality Assessments

Arugula leaves were evaluated for indices of leaf senescence (i.e., wilting, discolouration) and decay. Twenty leaves were subsampled from each antioxidant treatment/storage temperature replicate and were considered wilted if any part of the leaf surface area was flaccid. Leaf discolouration included evidence that any part of the leaf had one or more of the following: yellow, brown, and translucent tissues. Leaves were considered decayed if any part of the leaf was slimy. In all cases, the incidences of wilting, discolouration, and decay were based on the appearance of their respective symptoms, regardless of severity. The separate incidences of leaf discolouration, wilting, and decay were each calculated as the ratio of leaves exhibiting the respective symptoms relative to the total number of leaves evaluated multiplied by 100%. Leaf surface colour was determined with a portable Konica Minolta CR-400 Chroma Meter (Folio Instruments Inc., Kitchener, ON, Canada) equipped with a lens size of 8 mm. All colourimetry measurements were performed on the adaxial leaf surface. A total of 10 leaves (each having a blade width greater than 8 mm) were subsampled from each antioxidant treatment/storage temperature treatment replicate. Each leaf was assessed for the following colour parameters: lightness (L*), red-green (a*), and blue-yellow (b*). Lightness is a measure of colour saturation on a 100-point scale where 0 represents the darkest colour of black and 100 represents the lightest colour of white. The a* and b* parameters were used to calculate the colour attributes of hue angle and chroma using Equations (1) and (2), respectively:

(1) $$\mathrm{Hue}\;\mathrm{angle}=180+\tan^{-1}\frac{b^{*}}{a^{*}}$$

(2) $$\mathrm{Chroma}=\sqrt{{\left(a^{*}\right)}^{2}+{\left(b^{*}\right)}^{2}}$$

Hue angles represent different colours on a 360° scale, where 90° and 180° correspond to yellow and green, respectively. Chroma is an indicator of colour intensity, where higher chroma values denote increased colour saturation. The L*, a*, and b* parameters were used to determine the leaf browning index as developed by Buera et al. [33]. The browning index was calculated according to Equation (3):

(3) $$\mathrm{Browning}\;\mathrm{index}=[100\;(x-0.31)]/\;0.172\;;\;x=(a^{*}+1.75L^{*})\;/\;(5.645L^{*}+a^{*}-3.012b^{*})$$

At each sampling time, leaves were weighed immediately after sampling for the fresh weight determination. Thereafter, the leaves were placed in a drying oven for 10 days, and the dehydrated leaves were weighed to obtain the dry weight. The leaf water content of each sample was calculated by using Equation (4):

(4) Water content=((Fresh weight−Dry weight) / Fresh weight) × 100%

### 2.3. Metabolite Analyses

The frozen leaf material corresponding to each antioxidant treatment/storage temperature replicate was powdered with a mortar and pestle under liquid N_2_. The frozen leaf powder was used for the separate analyses of EGT, glutathione metabolites, ascorbate metabolites, chlorophylls and carotenoids as described below. Unless otherwise mentioned, all reagents were purchased from Sigma-Aldrich Canada (Oakville, ON, Canada). HPLC consumables were purchased from Agilent Technologies Canada (Mississauga, ON, Canada). All solvents and acids that were used for the metabolite analyses were purchased from Fisher Scientific Canada (Mississauga, ON, Canada).

#### 2.3.1. Chlorophyll and Carotenoid Determinations

Chlorophyll and carotenoid determinations were performed with minor modifications of established methods [34,35]. For each antioxidant treatment/storage temperature replicate, 36 mg subsample of frozen leaf powder was combined with 1.8 mL of prechilled methanol and vortexed for 1 min. The sample was left on ice for 5 min, vortexed, and then centrifuged at 14,800× *g* for 5 min at ambient temperature. The supernatant (or a 1.5- to 2-fold dilution) was transferred to a 1 mL quartz cuvette, and the chlorophyll and carotenoid concentrations were determined on a BioTek Epoch 2 (Fisher Scientific Canada) spectrophotometer by simultaneous measurement of absorbances at 470 nm, 652 nm, and 665 nm. The concentrations of chlorophyll *a*, chlorophyll *b*, and carotenoids within each arugula leaf sample were based on mathematical formula calculations for methanolic extracts as described previously [35] and were expressed on a fresh weight basis.

#### 2.3.2. HPLC Analysis of Leaf Ergothioneine

EGT analysis was performed as described previously [27] with minor modifications. Briefly, for each antioxidant treatment/storage temperature replicate, 0.5 g of frozen arugula leaf powder was combined with 20 volumes of ice-cold extraction solution (70% [*v*/*v*] ethanol containing 10 mM dithiothreitol and 100 μM betaine). The mixture was vortexed for 20 min and then combined with 4 volumes of 70% ethanol (*v*/*v*) containing 1% (*w*/*v*) sodium dodecyl sulfate. The mixture was inverted on a nutator for 10 min and then centrifuged at 1500× *g* for 20 min. The supernatant was passed through a 0.45 μm Restek™ polytetrafluoroethylene syringe filter (Fisher Scientific Canada). A 5 mL aliquot of the filtrate was concentrated using a SpeedVac. The dried residue was resuspended in 150 μL Milli-Q water and re-centrifuged at 14,800× *g* for 1 min. Thereafter, a 10 μL aliquot of the supernatant was injected onto a Kinetex^®^ hydrophilic interaction liquid chromatography column (5 μm, 100 Å, 150 mm × 4.6 mm) (Phenomenex Inc., Torrance, CA, USA) that was connected to an Agilent 1200 (or Agilent 1100) HPLC–DAD. The column was pre-equilibrated with acetonitrile: 20 mM ammonium acetate ([pH 6.0], 85:15, *v*/*v*; Solvent A) at a flow rate of 1 mL min^–1^ and maintained at 25 °C. EGT was eluted from the column with a gradient of solvent B (Milli-Q water: 20 mM ammonium acetate [pH 6.0], 85:15, *v*/*v*) as follows: 0–7 min (0% solvent B), 7–15 min (0–15.6% solvent B), 15–25 min (15.6–33.3% solvent B), and then held at 33.3% B for an additional 2 min. EGT was detected at 264 nm. To confirm the EGT retention time, duplicates of representative arugula samples were spiked with 3 nmol of an authentic EGT standard (Sigma-Aldrich Canada) prior to HPLC analysis. For the quantification of EGT, the peak area of each injected sample was compared to the known range of an authentic EGT standard (0.02 to 5 nmol). The leaf EGT concentration was expressed on a fresh weight basis and corrected for the percent recovery of authentic EGT (10 nmol) that was spiked into a duplicate sample at the point of extraction. 

#### 2.3.3. Spectrophotometric Analysis of Glutathione Metabolites via Enzyme-Coupled Assays 

The spectrophotometric detection of GSH and GSSG was performed with an enzyme-coupled assay [27]. All reagents were prepared fresh daily and kept on ice prior to the spectrophotometric analysis. For the extraction of total glutathione (i.e., sum of GSH and GSSG), 100 mg of frozen leaf powder was combined with 10 volumes of 200 mM HCl and vortexed for 5 min. The extract was centrifuged at 13,000× *g* for 10 min at 4 °C. A 0.5 mL aliquot of the supernatant was acidified with 0.2 M NaH_2_PO_4_ (pH 5.6) and then neutralised with 0.2 M NaOH. To assess the amount of GSSG in each leaf sample, a 200 μL aliquot of the neutralised extract was mixed with 1 μL of 2-vinylpyridine and inverted on a nutator for 60 min under darkness. The 2-vinylpyridine-treated extracts were centrifuged at 14,800× *g* for 10 min. For the determination of total glutathione, a 40 μL aliquot of the neutralised extract (or its dilution) was transferred to a well within a microplate and combined with 170 μL of assay mixture (118 mM NaH_2_PO_4_, 5.9 mM EDTA, 590 μM NADPH, 710 μM 5,5-dithio-bis-(2-nitrobenzoic acid)), which was prepared as described by Sivakumar and Bozzo [27]. The reaction was initiated by the addition of 200 milliunits of GSH reductase from *Saccharomyces cerevisiae*; the enzyme solution was prepared as described previously [27]. The rate of 5-thio-2-nitrobenzoic acid formation from the GSH-mediated reduction of 5,5-dithio-bis-(2-nitrobenzoic acid) was detected at 412 nm with a BioTek Epoch 2 spectrophotometer (Fisher Scientific Canada). For the determination of GSSG in each sample, the assay used 40 μL of the 2-vinylpyridine-treated extract (or a dilution thereof) in place of the original leaf extract. For each extract, the total glutathione and GSSG were quantified after comparing their rates of 5-thio-2-nitrobenzoic acid formation to those detected in assays that were performed with a known range of GSH (0.05 to 1 nmol) and GSSG (0.01 to 0.4 nmol) standards, respectively. Total glutathione and GSSG data were expressed on a fresh weight basis and corrected for the percent recoveries of authentic GSH (40 nmol) and GSSG (10 nmol) spikes that were added to representative replicate samples. The GSH within each arugula sample was calculated as the difference between total glutathione and GSSG. 

#### 2.3.4. HPLC Analysis of Leaf Ascorbate Metabolites 

Ascorbate analysis was performed as described previously [34,36], with minor modifications. For the analysis of each antioxidant treatment/storage temperature replicate, 200 mg of frozen leaf powder was transferred to a pre-chilled mortar containing 100 mg of acid-washed silica sand and 5 volumes of 6% (*w*/*v*) *meta*-phosphoric acid and homogenised for 50 s with a pre-chilled pestle. The extract was left on ice for 3 min under darkness and then clarified by centrifugation at 13,000× *g* for 10 min at 4 °C. The clarified extract was held on ice under darkness for 15 min prior to syringe filtration (0.45 μm Restek™ polytetrafluoroethylene). To determine the amount of ascorbate within each leaf extract, a 5 μL aliquot of the treated extract was analysed on a Restek™ Ultra Aqueous C18 column (5 μm, 100 Å, 150 mm × 4.6 mm) (Fisher Scientific Canada) attached to an Agilent 1100 HPLC–DAD system with the column temperature maintained at 20 °C. The column was pre-equilibrated in 20 mM *ortho*-phosphoric acid. Isocratic elution of ascorbate was performed at 1 mL min^–1^. For the determination of total ascorbate (i.e., sum of ascorbate and DHA), a 50 μL aliquot of the filtered extract was combined with 25 μL of 400 mM Tris base containing 200 mM dithiothreitol (DTT). DTT-treated leaf extracts were incubated at ambient temperature under darkness for 15 min, followed by the addition of 25 μL of 8.5% (*v*/*v*) *ortho*-phosphoric acid. A 5 μL aliquot of the DTT-treated extract was analysed by HPLC as described above, although the analysis period was extended to 28 min to ensure the DTT was fully eluted from the column. In both cases, ascorbate was detected at 254 nm, and the peak area and retention time were compared to a known range of an authentic ascorbate standard (0.1 to 7.5 nmol). All data were expressed on a fresh weight basis and corrected for the percentage recovery of ascorbate (i.e., between 150 and 400 nmol) that was spiked into a representative duplicate sample. The amount of DHA in each sample was calculated as the difference between the total ascorbate minus ascorbate. 

### 2.4. Statistical Analyses

All statistical analyses were performed with R version 4.3.0 [37]. The experimental design followed a randomised complete block design (RCBD) with repeated measures sampling. Prior to statistical analysis, assumptions of normality were assessed using the Shapiro–Wilk test and quantile–quantile (Q–Q) plot, while assumptions of homoscedasticity were assessed using Levene’s test. When required, data were log-transformed to satisfy assumptions of normality and homoscedasticity. To determine the effects of antioxidant treatment and storage period on arugula quality, data were analysed with a two-way ANOVA. Means were compared with Tukey’s HSD test and were considered statistically significant at *p* ≤ 0.05.

## 3. Results and Discussion

### 3.1. Effect of Antioxidant Dip Treatments and Storage Temperature on the Senescence and Quality of Arugula 

**Figure 1:**
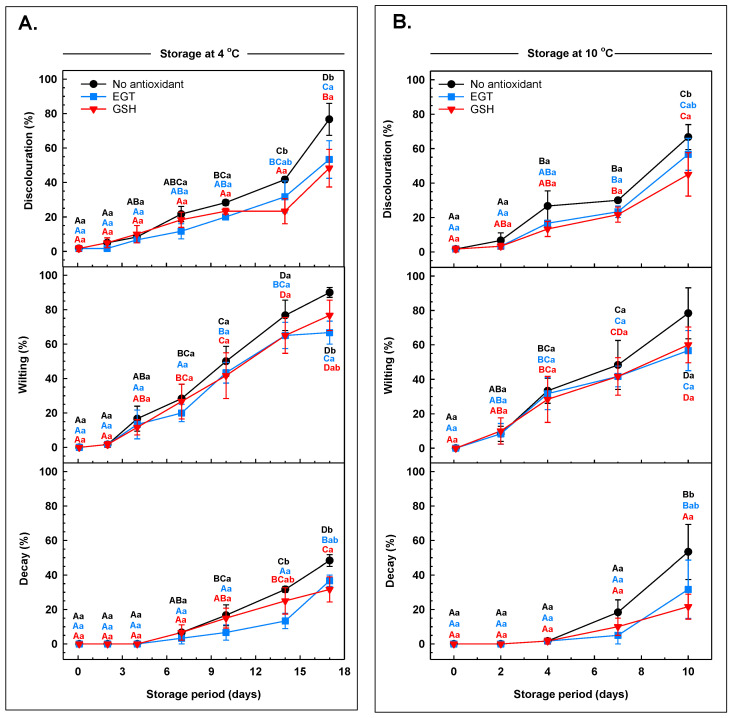
The incidence of discolouration, wilting, and decay in arugula immersed in a pre-storage dip containing either 100 μM ergothioneine (EGT), 500 μM glutathione (GSH), or no antioxidant, and then stored at 4 °C (**A**) or 10 °C (**B**). Each datum represents the mean incidence ± SE of three experimental replicates. Within each plot, uppercase letters denote statistical comparisons within a treatment across the storage period; lowercase letters denote statistical comparisons across the treatments at each postharvest sampling time. Shared letters represent no significant difference between means at *p* ≤ 0.05.

Arugula leaves treated with or without antioxidant dip solutions (i.e., EGT or GSH) were assessed for senescence and quality attributes at various times during their storage at 4 °C or 10 °C. These senescence and quality-related changes were more prevalent in non-antioxidant-treated leaves at both storage temperatures (Supplementary Information, Figures S1 and S2). A subjective analysis revealed a small incidence (i.e., between 2% and 22%) of leaf discolouration (i.e., leaf yellowing, browning, and/or translucency) within the initial 7 days of storage at 4 °C, regardless of treatment (Figure 1A). Thereafter, the incidence of discolouration was most apparent in the non-antioxidant-treated arugula, with most of the leaves discoloured by the end of the 17-day storage period. Overall, there was a smaller incidence of discolouration in the leaves of both antioxidant treatments. On day 14 of storage, GSH-treated arugula had 44% less discolouration than the incidence apparent in non-antioxidant-treated leaves. Thereafter, there was respectively 30% and 37% less discolouration in EGT-treated and GSH-treated leaves as compared to the non-antioxidant-treated leaves. Leaf discolouration was minimal over the initial two days of 10 °C storage and little affected by either antioxidant dip treatment (Figure 1B). On day 7 at this suboptimal temperature, 22% to 30% of the leaves were discoloured, regardless of the antioxidant treatment. Thereafter, nearly two-thirds of all leaves in the non-antioxidant treatment were discoloured, which approximated the incidence of discolouration in EGT-treated leaves but was 48% higher than in the GSH treatment. The limited discolouration of GSH-treated arugula fits with previous research showing that exogenous GSH limits browning of okra [23] and sliced lotus roots [24]. 

Minor incidences of leaf wilting were apparent over the initial 4 days of storage at 4 °C in non-antioxidant-treated arugula and the GSH treatment (Figure 1A). The incidence of wilting in EGT-treated leaves remained low and unchanged for an additional three days thereafter. Most of the non-antioxidant-treated leaves were wilted after 17 days at 4 °C. At this same time, a similar incidence of wilting was evident for GSH-treated leaves but was 26% smaller in leaves that were dipped in EGT prior to storage. Leaf wilting ranged from 42% to 48% within the first week of storage at 10 °C, regardless of whether antioxidants were applied prior to storage (Figure 1B). Thereafter, the incidence of leaf wilting was unchanged in EGT-treated and GSH-treated leaves, whereas a 62% increase occurred in the non-antioxidant treatment. Typically, wilting is associated with the loss of moisture, but there was no clear difference in the leaf water content of stored arugula across all antioxidant treatments (Supplementary Information, Figure S3). The similar amount of leaf water content across treatments may be because the arugula was packaged in clamshells, which tend to limit the loss of moisture. The absence of a change in the water content of arugula with storage mirrors the finding of Agüero et al. [38]. This group reported that the water content of bagged butterhead lettuce remained unchanged over 28 days at 0 to 2 °C. The wilting of arugula is likely due in part to increased electrolyte leakage with postharvest senescence. Medina et al. [39] noted that senescence and electrolyte leakage of baby spinach are exacerbated by low relative humidity in the storage atmosphere. Electrolyte leakage is a symptom of leaf senescence in cabbage, kale, lettuce, and spinach [40]. Exogenous EGT maintained the texture of button mushrooms and minimised electrolyte leakage during storage at 4 °C [32]. Interestingly, we found that immersion of arugula in EGT lessened the incidence of wilting at 4 °C. It is tempting to speculate that EGT may also limit electrolyte leakage in postharvest arugula. 

There was no evidence of decay throughout the initial 7 days of storage regardless of temperature (Figure 1), but decay symptoms were apparent thereafter. Pre-storage GSH and EGT dips delayed the increase in the incidence of decay in leaves stored at 4 °C until days 14 and 17, respectively. EGT and GSH reduced leaf decay by 58% and 34% on days 14 and 17 respectively, relative to the non-antioxidant treatment. By comparison, approximately half of the non-antioxidant-treated leaves had symptoms of decay after 10 days at 10 °C. A similar proportion of EGT-treated leaves were decayed by the end of the 10 °C storage period. Conversely, there was 59% less incidence of decay in the GSH treatment. The smaller incidence of leaf decay in GSH-treated arugula relative to the other treatments may be due in part to the ability of GSH to limit microbial spoilage. Ali et al. [24] demonstrated that exogenous GSH treatments minimised bacterial, yeast, and mould counts in sliced lotus root. Similarly, chitosan coatings enriched with GSH limit bacterial growth in beef and shellfish products [41,42]. Additional research is required to determine whether exogenous GSH controls specific food-borne pathogens during postharvest storage of arugula.

**Figure 2:**
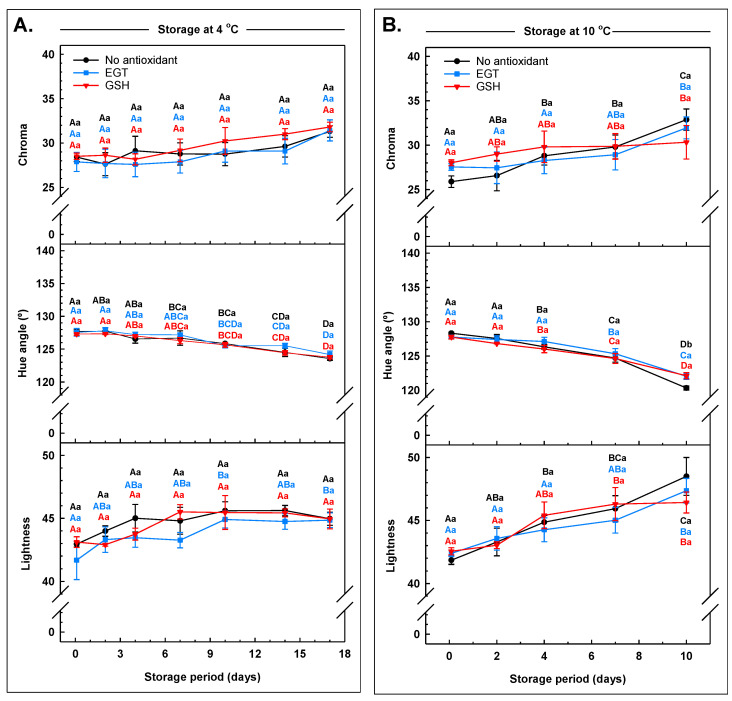
Leaf chroma, hue angle, and lightness in arugula that was immersed in a pre-storage dip containing either 100 μM ergothioneine (EGT), 500 μM glutathione (GSH), or no antioxidant, and then stored at 4 °C (**A**) or 10 °C (**B**). Each datum represents the mean ± SE of three experimental replicates. Within each plot, uppercase letters denote statistical comparisons within a treatment across the storage period; lowercase letters denote statistical comparisons across the treatments at each postharvest sampling time. Shared letters represent no significant difference between means at *p* ≤ 0.05.

Colourimetry determined that leaf chroma was stable throughout the storage period at 4 °C (Figure 2A). Conversely, chroma was increased in the non-antioxidant-treated leaves after 2 days at 10 °C (Figure 2B). For both antioxidant treatments, leaf chroma was stable for the initial week of storage at this temperature but then increased thereafter. At both temperatures, there was no impact of either antioxidant treatment on leaf chroma. Leaf hue angle was also assessed for the stored arugula leaves. The leaf hue angles detected across all antioxidant treatments and storage temperatures were between 90° and 180°, which represent yellow and green colouration, respectively. Leaf hue angle declined by as much as 4° over the complete 4 °C storage period, but there was no impact of the pre-storage antioxidants, implying the same degree of yellowing across all three treatments (Figure 2A). Conversely, a greater and sharper decrease in the leaf hue angle was apparent in arugula stored at 10 °C (Figure 2B). The hue angle decreased by 8° in the non-antioxidant-treated leaves held at 10 °C. Thus, in non-antioxidant-treated leaves, yellowing was more pronounced at 10 °C relative to leaves held at 4 °C. This fits with previous findings that arugula yellowing is more prominent at 10 °C than at lower storage temperatures [6]. Similar trends have been described for other leafy green vegetables such as pak choi and romaine lettuce [43,44]. A decline in the leaf hue angle was not apparent in EGT-treated leaves until day 7 of storage at 10 °C. By the end of the 10-day storage period, leaf hue angle was nearly 2° greater in the EGT and GSH treatments relative to the non-antioxidant-treated leaves. Leaf lightness remained unchanged with storage at 4 °C, except for the EGT-treated leaves. For this treatment, lightness increased 8% by the tenth day of storage and was unchanged thereafter. A 7.2% increase in leaf colour lightness was evident in non-antioxidant-treated leaves by the fourth day of storage at 10 °C. An additional 8% increase was apparent after day 4.

**Figure 3:**
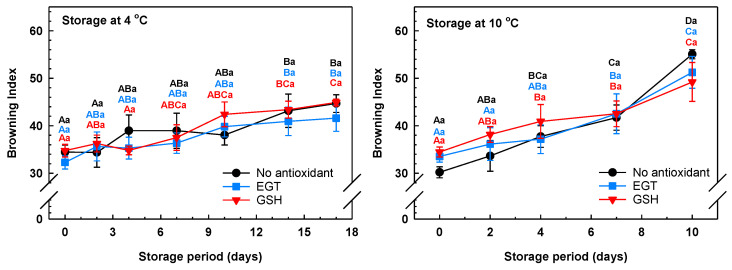
Browning index of arugula immersed in a pre-storage dip containing either 100 μM ergothioneine (EGT), 500 μM glutathione (GSH), or no antioxidant, and then stored at 4 °C or 10 °C. Each datum represents the mean ± SE of three experimental replicates. Within each plot, uppercase letters denote statistical comparisons within a treatment across the storage period; lowercase letters denote statistical comparisons across the treatments at each postharvest sampling time. Shared letters represent no significant difference between means at *p* ≤ 0.05.

The colourimetric parameters of L*, a*, and b* were also used to calculate the browning index of the antioxidant-treated arugula leaves. There was an increase in the browning index of antioxidant and non-antioxidant-treated leaves during storage, although an overall greater browning index was evident by the end of the storage period at 10 °C than at 4 °C (Figure 3). Browning was not apparent at 4 °C until day 14 of storage. The delayed browning response at 4 °C fits with a previous report that less than 1% of the surface area of arugula leaves browned after 3 days at 4 °C [45]. The postharvest browning of horticultural crops is correlated with an increase in polyphenol oxidase activity that occurs with cellular collapse [17]. Polyphenol oxidases are ubiquitous across plants and use a variety of phenolic acids and polyphenols as substrates in the presence of O_2_ to form reactive *o*-quinones that readily react with amino acids and proteins to yield the brown pigment melanin [46]. Interestingly, browning and polyphenol oxidase activity are more prominent in low-ascorbate salad vegetables (e.g., lettuce and endive) relative to arugula that contains greater concentrations of this antioxidant [45,47]. Although there was no impact of EGT or GSH on the browning index of the arugula at one or more timepoints of the storage at 10 °C, an increase in the index occurred by day 4 for the non-antioxidant and GSH treatments. Conversely, the browning index was not altered in EGT-treated leaves until the seventh day of 10 °C storage. This fits with the capacity of EGT to control browning of seafood, fish, and beef [28,29,30], button mushroom [32], and fermented soybean [48]. Qian et al. [32] noted that application of EGT controlled melanosis in mushrooms through inhibition of polyphenol oxidase. Similar mechanisms may explain the reduction of browning events associated with the discolouration of stored arugula leaves. Overall, there was little overlap between how the antioxidant treatments impacted the incidence of leaf discolouration and the browning index. These phenomena could be due to inconsistencies between the subjective assessment of whole leaf discolouration, including yellowing, browning, and their colourimetric detection. The colourimeter assesses a smaller area of the leaf and may not have accounted for the leaf yellowing and browning of the whole arugula leaf, including those respective events occurring at the leaf tips and edges. Batziakas et al. [49] proposed a similar argument for the lack of a correlation between the decline in hue angle of senescing spinach and total leaf chlorophyll losses, as the latter involves extraction of pigments from whole leaves.

**Figure 4:**
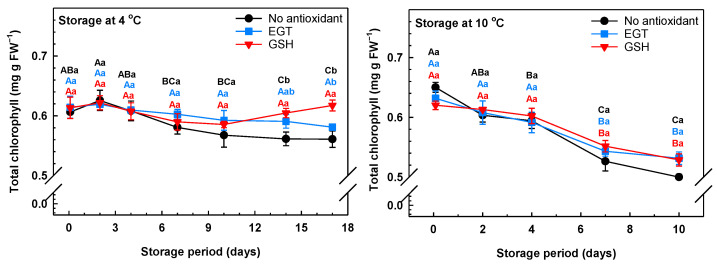
Total chlorophyll in arugula immersed in a pre-storage dip containing either 100 μM ergothioneine (EGT), 500 μM glutathione (GSH), or no antioxidant, and then stored at 4 °C or 10 °C. All total chlorophyll concentrations are expressed on a fresh weight (FW) basis. Each datum represents the mean ± SE of three experimental replicates. Within each plot, uppercase letters denote statistical comparisons within a treatment across the storage period; lowercase letters denote statistical comparisons across the treatments at each postharvest sampling time. Shared letters represent no significant difference between means at *p* ≤ 0.05.

Postharvest yellowing of green vegetables is linked to chlorophyll degradation [50]. In our study, chlorophyll losses in arugula were more apparent at 10 °C than at 4 °C (Figure 4). Similarly, temperature abuse (e.g., non-refrigerated conditions) promotes chlorophyll losses in arugula of *E. sativa* [6], broccoli [51], cabbage [52], purslane [53], and spinach [54]. The possibility remains that the greater loss of arugula chlorophyll at 10 °C is associated with higher activities of enzymes and/or upregulation of genes associated with the chlorophyll catabolism pathway. Chlorophyll turnover in plants requires the sequential action of the following enzymes: Mg-dechelatase, pheophytinase, pheophorbide *a* oxygenase, and red chlorophyll catabolite reductase [55]. In addition, the disappearance of chlorophylls during postharvest senescence of broccoli is associated with the increased expression of chlorophyll turnover genes, with both phenomena being more prominent at the warm storage temperature of 20 °C than at lower temperatures [56].

The separate pre-storage applications of EGT and GSH preserved total chlorophyll concentrations in arugula leaves during storage at 4 °C (Figure 4). Conversely, a 10% loss of the total chlorophyll concentration was apparent in the non-antioxidant-treated leaves by day 14 of storage. By the end of the 17-day storage period, the total chlorophyll concentration in GSH-treated leaves was 6% and 10% greater than that of EGT and non-antioxidant treatments, respectively. In GSH-treated leaves, there was respectively 7% and 10% more chlorophyll *a* and chlorophyll *b* on day 14 of 4 °C storage relative to non-antioxidant-treated leaves (Supplementary Information, Figure S4). These differences were maintained thereafter. No change in the total chlorophyll concentration was evident in both antioxidant treatments over the first 4 days of storage at 10 °C, whereas a 9% decline occurred in non-antioxidant-treated leaves (Figure 4). Conversely, the chlorophyll *a* concentration was unchanged between day 0 and day 4 of 10 °C in GSH-treated leaves but not in the other treatments (Supplementary Information, Figure S4). This disparity in the impact of the antioxidants on the concentrations of total chlorophyll and chlorophyll *a* in arugula stored at 10 °C may be due in part to the spectrophotometric analysis used in this study. Schoefs has argued that spectrophotometric analysis of chlorophylls within crude plant extracts is not as sensitive as HPLC-based separation and detection of the individual chlorophyll and carotenoid species [57]. HPLC–MS analysis determined that colourless non-fluorescent tetrapyrroles are formed from chlorophyll molecules in the de-greening of broccoli heads during storage at 22 °C for 5 days [58]. It would be interesting to determine whether chlorophyll degradation products occur in senescing arugula.

Leaf chlorophyll *a* concentration decreased by as much as 27% by the end of the storage period, although there was no impact of the antioxidant treatments. Chlorophyll *b* concentration decreased 10% in non-antioxidant-treated leaves after 10 days at 10 °C. By comparison, chlorophyll *b* concentrations were unchanged within any of the antioxidant-treated leaves (Supplementary Information, Figure S4). Similarly, exogenous GSH maintains the chlorophyll status of okra stored at 10 °C [23]. It is well known that there is little chlorophyll biosynthesis in postharvest vegetables. In fact, Luo et al. [59] determined there was a decrease in the transcript levels for various chlorophyll biosynthesis genes in broccoli heads held at 10 °C for 12 days, whereas the converse trend was evident for the transcripts of chlorophyll degradation enzymes. Broccoli florets treated with melatonin (an oxidative stress-reducing molecule) undergo little chloroplast breakdown and have lower gene expression and activities for chlorophyll degradation enzymes relative to untreated broccoli [60]. It is tempting to speculate that exogenous GSH preserves the ultrastructure and membrane composition of chloroplasts within arugula leaves during postharvest storage, thereby limiting chlorophyll degradation. 

Like chlorophylls, the concentrations of carotenoids also decline with senescence in various vegetable commodities [61,62,63]. The total carotenoid concentrations within the arugula leaves (Supplementary Information, Figure S5) were stable with storage at 4 °C, regardless of the pre-storage antioxidant treatments. However, the antioxidant treatments stabilised the total carotenoid concentrations in arugula stored at 10 °C. By comparison, a 10% loss of total carotenoids occurred in the non-antioxidant-treated leaves. By the end of the storage period, the total carotenoid concentration was 10% greater in GSH-treated leaves than in the non-antioxidant treatment. This fits with a report that EGT-enriched extracts from branching oyster mushrooms limit the oxidation of the algal carotenoid astaxanthin within artificial liposomes [64]. With respect to the benefit of GSH on carotenoid composition in stored arugula, this may be due to its sequestration of ROS that would otherwise damage chloroplasts and carotenoids. In fact, the foliar application of GSH limits oxidative stress and increases the carotenoid concentrations of rice plants during submergence, an abiotic stress associated with ROS accumulation [65].

### 3.2. Effect of Antioxidant Dip Treatments and Storage Temperature on Antioxidant Profiles of Arugula Leaves

**Figure 5:**
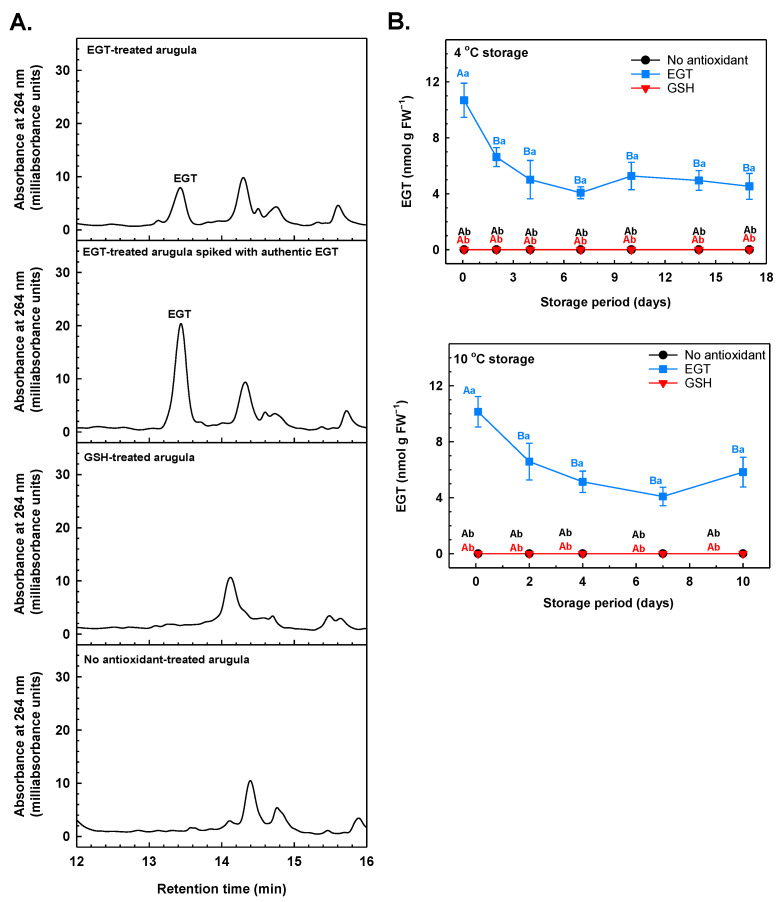
Ergothioneine (EGT) in arugula immersed in a pre-storage dip containing either 100 μM EGT, 500 μM glutathione (GSH), or no antioxidant, and then stored at 4 °C or 10 °C. (**A**) HPLC–DAD analysis of EGT in extracts prepared from arugula leaves sampled on day 0 of 4 °C storage and within 2 h of their exposure to dip solutions. Chromatograms represent arugula leaves treated with 100 µM EGT prior to storage (top chromatogram); arugula leaves treated with 100 µM EGT prior to storage, and the extract spiked with 3 nmol EGT at the point of HPLC analysis (second chromatogram from top); arugula leaves treated with 500 µM GSH prior to storage (third chromatogram from top); and arugula leaves not treated with antioxidants prior to storage (bottom chromatogram). (**B**) Alterations in EGT concentrations in arugula leaves as a function of storage period at 4 °C or 10 °C after a pre-storage dip treatment with or without antioxidants. All EGT concentrations are expressed on a fresh weight (FW) basis. Each datum represents the mean ± SE of three experimental replicates. Within each plot, uppercase letters denote statistical comparisons within a treatment across the storage period; lowercase letters denote statistical comparisons across the treatments at each postharvest sampling time. Shared letters represent no significant difference between means at *p* ≤ 0.05.

The effects of exogenous EGT on the quality of mushrooms and meat products (e.g., shrimp, beef, and fish) have been tested previously [28,30,32], but these studies did not assess whether EGT was present within these foods after its application. In our study, the concentrations of EGT and GSH dipping solutions were confirmed before and after arugula treatment. These analyses confirmed that the antioxidants had not degraded within the dip solution during the 30 min incubation period. HPLC analysis revealed the presence of EGT (retention time = 13.4 min) in ethanolic extracts of EGT-treated leaves that were sampled within 2 h of the dip treatment (Figure 5A). The retention time matched that of an authentic EGT standard that was spiked into an ethanolic leaf extract that was prepared for HPLC analysis. The occurrence of EGT in plants such as wheat is due to EGT uptake from the soil or through root–mycorrhizal associations [66]. EGT is not biosynthesised in plants [26]. Similarly, HPLC–DAD analysis revealed the absence of EGT within arugula sampled from the non-antioxidant and GSH treatments, regardless of storage temperature. After exposure to the 100 µM EGT dip, the greatest EGT concentration was detected in arugula leaves that were sampled at the beginning of the storage period (Figure 5B). EGT concentrations within the EGT-treated leaves decreased by 38% and 35% after 2 days at 4 °C and 10 °C, respectively. At both temperatures, EGT concentrations remained unchanged thereafter. In enoki and shiitake mushrooms, endogenous EGT concentrations tend to decline with prolonged refrigeration at 5 °C [67,68]. EGT degradation in mushrooms and in EGT-treated arugula is likely due to oxidation by divalent metal cations and ROS. In vitro analyses determined that EGT interacts with divalent metal cations (e.g., Fe^2+^), which may limit metal-associated oxidative damage in cells [69]. Additionally, in vitro studies established that H_2_O_2_ oxidises EGT to hercynine [70]. Other oxidation products of EGT include EGT disulfide and 5-*oxo*-EGT [71]. EGT is recycled from these EGT oxidation products in the presence of GSH and GSH reductase [71,72]. It is reasonable to speculate that EGT recycling may occur in EGT-treated arugula at the expense of the available GSH pool, which may explain the stable EGT status throughout much of the storage period.

**Figure 6:**
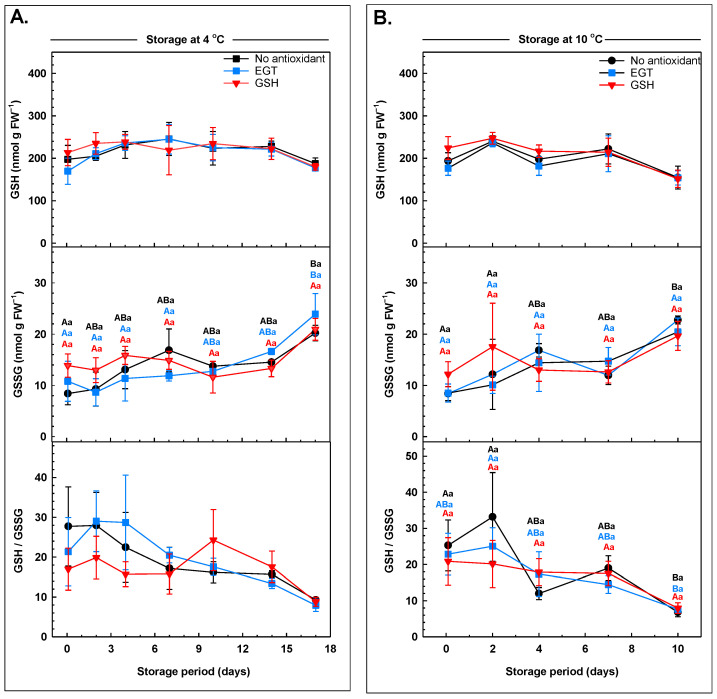
Glutathione profiles (glutathione, GSH; glutathione disulfide, GSSG; and GSH/GSSG ratio) in arugula immersed in a pre-storage dip containing either 100 μM ergothioneine (EGT), 500 μM glutathione (GSH), or no antioxidant, and then stored at 4 °C (**A**) or 10 °C (**B**). All glutathione metabolite concentrations are expressed on a fresh weight (FW) basis. Each datum represents the mean ± SE of three experimental replicates. Within each plot, uppercase letters denote statistical comparisons within a treatment across the storage period; lowercase letters denote statistical comparisons across the treatments at each postharvest sampling time. Shared letters represent no significant difference between means at *p* ≤ 0.05. Plots devoid of statistical lettering represent no significant differences across treatments or their sampling days.

The total glutathione and GSH concentrations in arugula were unchanged with storage, regardless of the type of antioxidant dip treatment (Figure 6; Supplementary Information, Figure S6). GSH-treated arugula did not contain higher levels of GSH on day 0 of storage relative to EGT-treated and non-antioxidant-treated leaves (Figure 6). Similarly, finite GSH levels were not enhanced in okra seed pods and mango fruit immediately after they were supplied with exogenous GSH [23,73]. There was no change in the total glutathione and GSH concentrations throughout the whole storage period at either storage temperature (Figure 6; Supplementary Information, Figure S6). Overall, there was an effect of the pre-storage antioxidant dips on the GSSG concentrations as a function of storage period. There was no change in the GSSG concentration of GSH-treated leaves across the 4 °C storage period. Conversely, GSSG increased 140% and 101% in the non-antioxidant treatment and the EGT treatment, respectively, by day 17 at 4 °C storage. In the non-antioxidant-treated leaves, the GSSG concentration increased 87% between day 2 and day 10 at 10 °C storage. There was no change in the GSSG concentrations in the leaves of either antioxidant treatment stored at 10 °C. The glutathione redox ratio (GSH/GSSG) was stable at 4 °C, regardless of antioxidant treatment. The GSH/GSSG ratio was unchanged for the first two days of storage at 10 °C, but by day 10 this ratio decreased 70% and 79% in the EGT and the non-antioxidant-treated leaves, respectively. By contrast, the GSH/GSSG ratio was stable in GSH-treated arugula at this warmer temperature. 

Assuming that GSH was absorbed by arugula leaves at the same efficiency as EGT, several biochemical routes for the absorbed GSH are likely. One possibility is that the exogenous GSH was quickly metabolised through the ascorbate-GSH recycling pathway. Yao et al. [22] noted the rapid upregulation of *DHAR1* in GSH-sprayed bell peppers at the onset of storage at 4 °C. Similarly, greater DHAR and GSH reductase activities were evident in GSH-treated lotus root slices stored at 5 °C relative to their untreated counterparts [24]. It is possible that the application of exogenous GSH enhanced the expression of arugula DHAR and GSH reductase genes and their enzyme activities. This may partially explain the stable GSSG concentrations in the stored arugula investigated in this study. Similarly, Li et al. [23] determined that exogenous GSH prevented the accumulation of GSSG in postharvest okra. It is known that GSSG accumulates with senescence in cherries, Chinese flowering cabbage, and lotus root [19,24,74]. Thus, the maintained GSSG concentration in GSH-treated arugula appears to be associated with the delayed yellowing and hence senescence at the warmer storage temperature, as well as the reduced incidence of leaf discolouration and decay at both storage temperatures investigated in this study. EGT stabilisation of arugula GSSG concentrations at 10 °C implies that there is little oxidation of GSH in EGT-treated arugula. Kalaras et al. [75] found a high degree of correlation between EGT and GSH concentrations in various culinary mushrooms. Typically, a decline in the glutathione redox status (i.e., GSH/GSSG ratio) occurs with the senescence of horticultural commodities, as demonstrated for Chinese flowering cabbage and pear fruit [19,36]. Although GSH/GSSG ratios remained stable in GSH-treated arugula at 10 °C, decreased GSH/GSSG ratios occurred with storage in the EGT and non-antioxidant treatments. The decline in the glutathione redox status with temperature abuse is likely due to decreased GSH reductase gene expression and activity. These phenomena are known to occur within postharvest commodities, including broccoli, when held under non-refrigerated storage temperatures [76,77,78]. 

**Figure 7:**
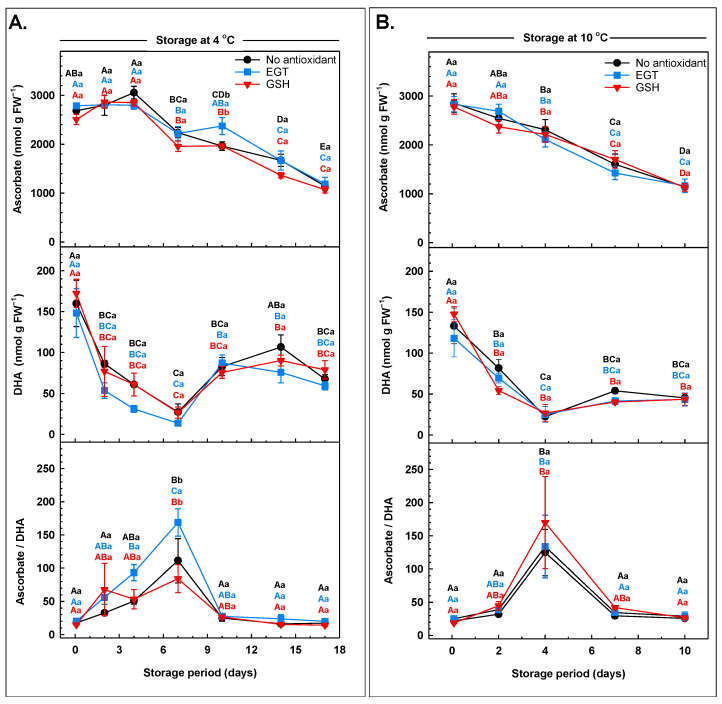
Ascorbate profiles (ascorbate, dehydroascorbate, DHA; and ascorbate/DHA ratio) in arugula immersed in a pre-storage dip containing either 100 μM ergothioneine (EGT), 500 μM glutathione (GSH), or no antioxidant, and then stored at 4 °C (**A**) or 10 °C (**B**). All ascorbate metabolite concentrations are expressed on a fresh weight (FW) basis. Each datum represents the mean ± SE of three experimental replicates. Within each plot, uppercase letters denote statistical comparisons within a treatment across the storage period; lowercase letters denote statistical comparisons across the treatments at each postharvest sampling time. Shared letters represent no significant difference between means at *p* ≤ 0.05.

Ascorbate degradation occurs with postharvest storage of pak choi, spinach, and watercress [79,80]. We also found that the total ascorbate concentrations in arugula decreased with storage, regardless of the antioxidant dip treatments applied prior to storage and the final storage temperature (Supplementary Information, Figure S7). Overall, total ascorbate concentrations decreased by approximately 60% at both temperatures, although these losses were evident on day 10 of storage at 10 °C and 7 days later for leaves held at 4 °C. Similarly, temperature abuse promotes ascorbate degradation in lettuce and broccoli [81,82]. This increased ascorbate degradation in arugula at 10 °C is likely due to increased ascorbate peroxidase activity, which has been shown for sweet pepper fruit stored at 25 °C [83]. Both ascorbate and DHA concentrations decreased with storage (Figure 7). There was no effect of either pre-storage antioxidant dip treatment on ascorbate metabolism within arugula stored at 10 °C. Conversely, ascorbate was 20% greater in the EGT treatment on the tenth day of storage at 4 °C relative to all other treatments. The ascorbate concentration decreased 31% in non-antioxidant-treated leaves after day 14 at 4 °C, whereas both antioxidant treatments limited the loss of ascorbate during this time. For both storage environments, DHA declined over the first 7 days, but the magnitude of these changes was greater at 4 °C than at 10 °C, regardless of antioxidant treatment. At the lower temperature, the DHA concentration was reduced 83% to 91% by day 7 of storage but increased by as much as 4.5-fold thereafter. In contrast, the DHA concentration declined 60% to 73% by day 7 at 10 °C but remained unchanged thereafter. The loss of DHA with storage is consistent with the near-complete disappearance of the DHA pool in arugula after 16 days at 5 °C [84]. DHA degradation in arugula likely culminates in the production of the catabolite oxalate. Dewhirst et al. [79] demonstrated that radiolabelled forms of DHA and oxalate were formed from [^14^C]-ascorbate that was supplied to postharvest spinach leaves. 

The concentrations of ascorbate and DHA within each antioxidant treatment/storage temperature replicate were used to calculate the corresponding ascorbate/DHA ratio. A 5.3-fold spike in the ascorbate/DHA ratio was evident in non-antioxidant-treated leaves on day 7 of 4 °C storage. A similar ascorbate/DHA ratio occurred in GSH-treated leaves at this time, whereas 52% more of this ratio was apparent in EGT-treated leaves. In all cases, the ascorbate/DHA ratio declined to pre-storage levels by day 10 of storage and remained unchanged thereafter. For leaves stored at 10 °C, there was also a transient 4.6-fold to 8-fold spike in the ascorbate/DHA ratio by the fourth day of storage, but there was no impact of the antioxidant dip treatments. The fluctuations in the ascorbate/DHA ratio mirror those reported for pak choi leaves stored at 20 °C [80]. The greater ascorbate redox status in EGT-treated arugula is consistent with the preservation of ascorbate in EGT-treated button mushrooms [32]. The possibility remains that EGT readily scavenges ROS in the stored arugula, thereby reducing the interactions between ascorbate and ROS. Alternatively, it may be that the absorbed EGT was recycled in the presence of ascorbate. We postulate that this would involve the Foyer–Halliwell–Asada pathway or a similar pathway that is dependent upon reducing power equivalents and reductases that can use oxidised forms of EGT and ascorbate.

## 4. Conclusions

Our investigation established that the shelf life of arugula *D. tenuifolia* ‘Letizia’ was extended by the pre-storage applications of GSH and EGT dip solutions. The shelf life of leafy green vegetables is linked to senescence-related yellowing in arugula [6,14] cabbage [52], green lettuce [85], and spinach [54]. The end of the shelf-life period for arugula is the point at which more than 50% of the leaves are senescent or decayed [9]. Using this criterion, in our study, the non-antioxidant-treated arugula had acceptable quality up to 14 days at 4 °C and up to 7 days at 10 °C. In comparison, the exogenous antioxidant applications investigated in our study extended shelf life by 3 days, with less than 50% of their leaves discoloured and decayed after 17 days at 4 °C. Exogenous GSH may extend shelf life at 10 °C beyond 10 days, as less than 50% of GSH-treated leaves were discoloured or decayed at this time. Given the reduction in browning with the pre-storage EGT dip treatment, this technology should also be tested for vegetables that are highly susceptible to postharvest browning (e.g., lettuce). The delay in the loss of arugula quality was linked to alterations in endogenous antioxidant profiles that were somewhat varied between the exogenous EGT and GSH treatments. Ascorbate degradation in EGT-treated leaves occurred at a slower rate than non-antioxidant-treated arugula at 4 °C. At both temperatures, these same leaves maintained their absorbed EGT after an initial decline. Finally, GSSG tends to accumulate with storage of leafy green vegetables, but this was not the case in the arugula leaves of both antioxidant treatments stored under the temperature abuse condition of 10 °C. This may be a consequence of increased recycling of GSSG by GSH reductase, which requires further investigation. In summary, the EGT and GSH antioxidant dip strategies that were developed in this study are promising for the shelf-life extension of arugula in commercial operations, including the retail sector. It is worth noting that for commercial purposes, horticultural products containing exogenous antioxidants must also satisfy regulatory and safety requirements for human consumption. Currently, the European Food Standards Agency has deemed that the addition of synthetic ergothioneine (i.e., Ergoneine^®^) to food products such as milk is safe for human consumption [86], but to the best of our knowledge, this antioxidant has not yet been regulated for use on fresh horticultural products.

## Data Availability

The data presented in this study are available in the results section of this article.

## Supplementary Materials

The following supporting information can be downloaded at: https://www.mdpi.com/article/10.3390/antiox13091140/s1, Figure S1: Senescence symptoms of arugula leaves after 17 days of storage at 4 °C; Figure S2: Senescence symptoms of arugula leaves after 10 days of storage at 10 °C; Figure S3: Water content in arugula immersed in a pre-storage dip containing either 100 μM ergothioneine (EGT), 500 μM glutathione (GSH), or no antioxidant, and then stored at 4 °C or 10 °C; Figure S4: Chlorophyll *a* and *b* concentrations in arugula immersed in a pre-storage dip containing either 100 μM ergothioneine, 500 μM glutathione, or no antioxidant, and then stored at 4 °C (A) or 10 °C (B); Figure S5: Total carotenoid concentrations in arugula immersed in a pre-storage dip containing either 100 μM ergothioneine, 500 μM glutathione, or no antioxidant, and then stored at 4 °C or 10 °C; Figure S6: Total glutathione concentrations in arugula immersed in a pre-storage dip containing either 100 μM ergothioneine, 500 μM glutathione, or no antioxidant, and then stored at 4 °C or 10 °C: Figure S7: Total ascorbate concentrations in arugula immersed in a pre-storage dip containing either 100 μM ergothioneine, 500 μM glutathione, or no antioxidant, and then stored at 4 °C or 10 °C.
