# Supplementary material for: Exogenous Ergothioneine and Glutathione Limit Postharvest Senescence of Arugula
Source: Antioxidants (Basel). 2024 Sep 21;13(9):1140. doi: 10.3390/antiox13091140 (PMC11429341; doi:10.3390/antiox13091140)
Supplement: Supplementary file 1 [file antioxidants-13-01140-s001.zip › antioxidants-3154465-supplementary.pdf]

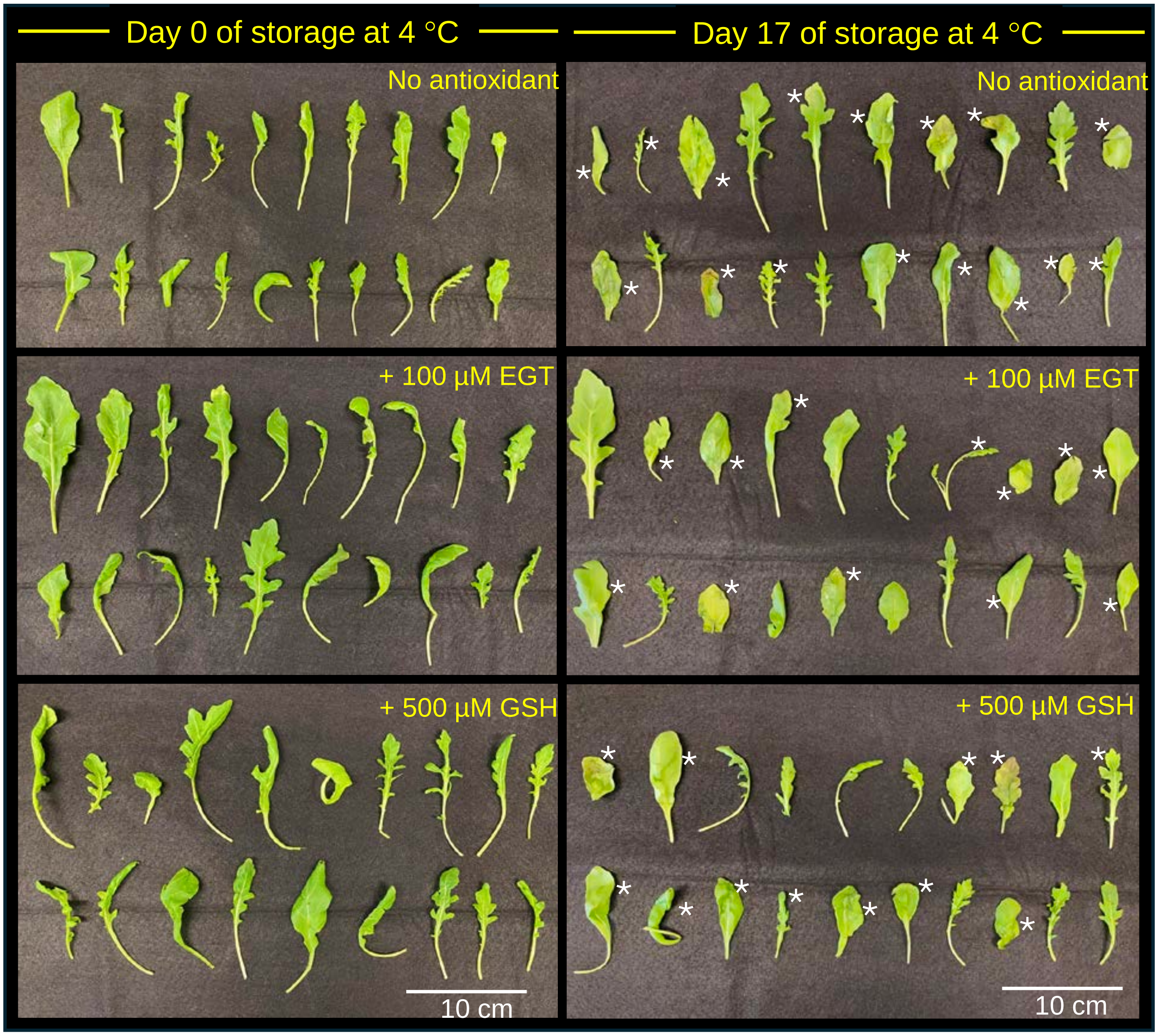

Figure S1. Senescence symptoms of arugula leaves after 17 days of storage at 4 °C. Arugula leaves were immersed in a pre-storage dip containing either 100 µM ergothioneine (EGT), 500 µM glutathione (GSH), or no antioxidant, and then stored at 4°C for 17 days. All images are from the same experiment. On the left side and right side are leaves sampled from all three antioxidant treatments on day 0 and day 17 of storage, respectively. Leaves proximal to a white asterisk denote those leaves that had symptoms of senescence and/or quality loss (e.g., one or more of yellowing, browning, translucency, wilting). Scale-bar = 10 cm.

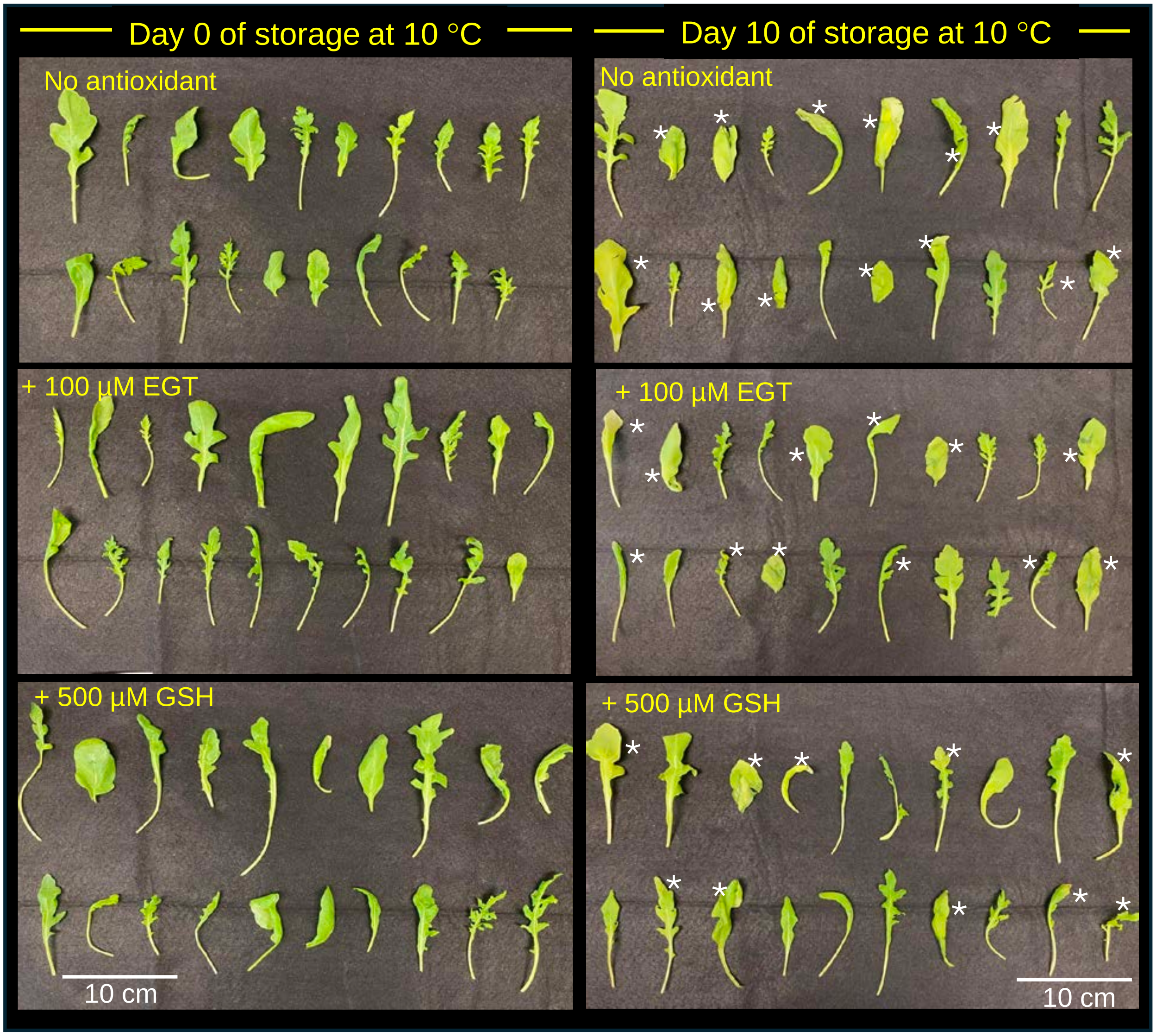

Figure S2. Senescence symptoms of arugula leaves after 10 days of storage at 10 °C. Arugula leaves were immersed in a pre-storage dip containing either 100 µM ergothioneine (EGT), 500 µM glutathione (GSH), or no antioxidant, and then stored at 10°C for 10 days. All images are from the same experiment. On the left side and right side are leaves sampled from all three antioxidant treatments on day 0 and day 10 of storage, respectively. Leaves proximal to a white asterisk denote those leaves that had symptoms of senescence and/or quality loss (e.g., one or more of yellowing, browning, translucency, wilting). Scale-bar = 10 cm.

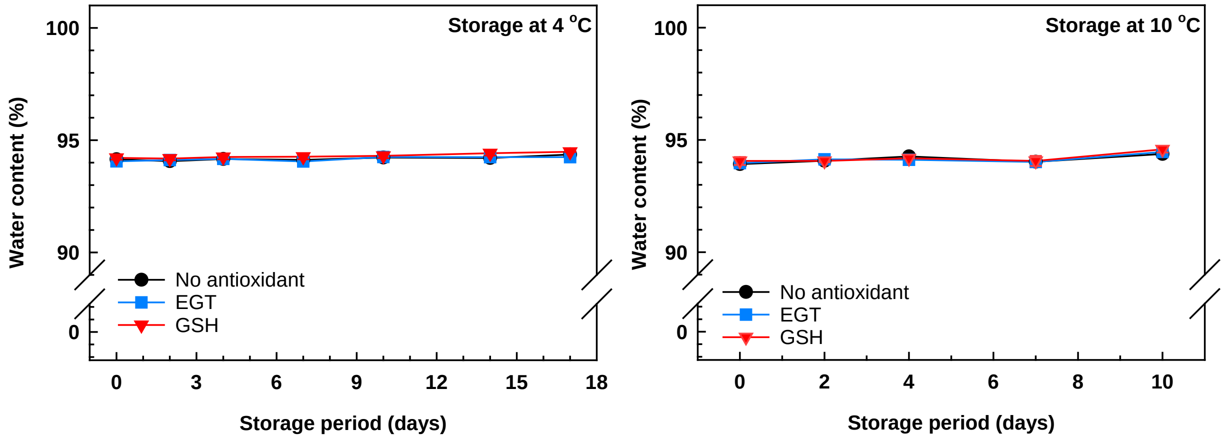

Figure S3. Water content in arugula immersed in a pre-storage dip containing either 100 µM ergothioneine (EGT), 500 µM glutathione (GSH), or no antioxidant, and then stored at 4 °C or 10 °C.

Each datum represents the mean water content ± SE of three experimental replicates. Plots devoid of statistical lettering represent no significant differences across treatments or their sampling days ($p \leq 0.05$).

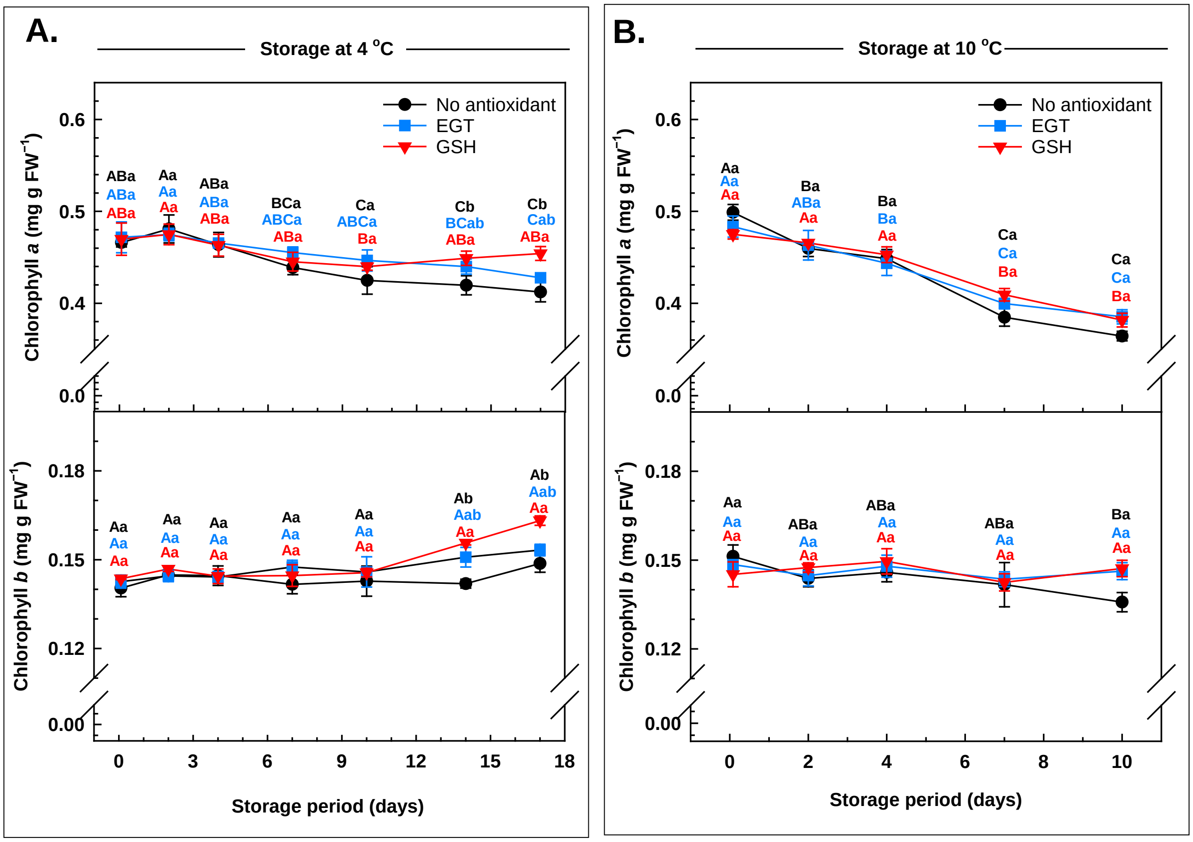

Figure S4. Chlorophyll *a* and *b* concentrations in arugula immersed in a pre-storage dip containing either 100 µM ergothioneine, 500 µM glutathione, or no antioxidant, and then stored at 4 °C (**A**) or 10 °C (**B**).

All data are expressed on a fresh weight basis. Each datum represents the mean chlorophyll concentration ± SE of three experimental replicates. Within each plot, uppercase letters denote statistical comparisons within a treatment across the storage period; lowercase letters denote statistical comparisons across the treatments at each postharvest sampling time. Shared letters represent no significant difference between means at $p \leq 0.05$.

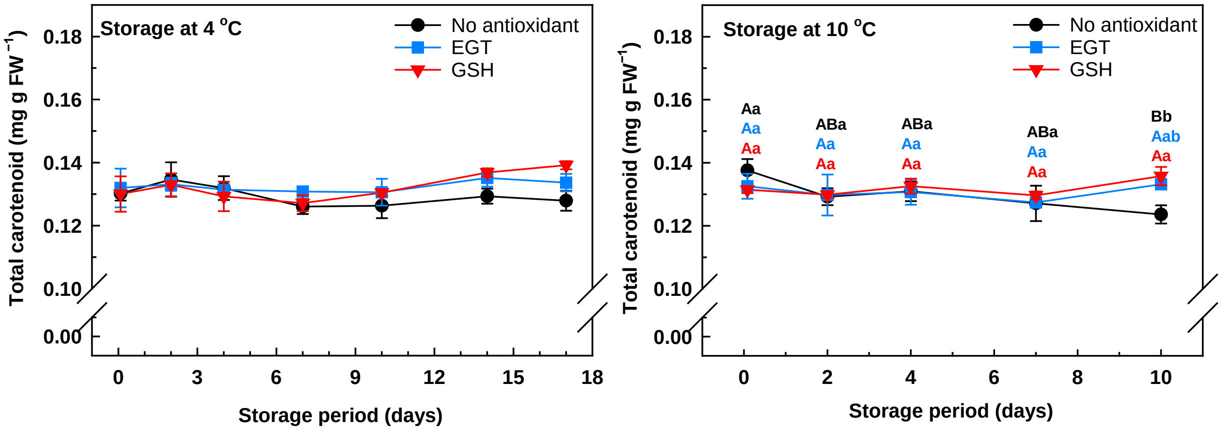

Figure S5. Total carotenoid concentrations in arugula immersed in a pre-storage dip containing either 100 μM ergothioneine, 500 μM glutathione, or no antioxidant, and then stored at 4 °C or 10 °C.

All data are expressed on a fresh weight basis. Each datum represents the mean total carotenoid concentration ± SE of three experimental replicates. Within each plot, uppercase letters denote statistical comparisons within a treatment across the storage period; lowercase letters denote statistical comparisons across the treatments at each postharvest sampling time. Shared letters represent no significant difference between means at $p \leq 0.05$. The 4 °C storage plot is devoid of statistical lettering as there were no significant differences across treatments or their sampling days

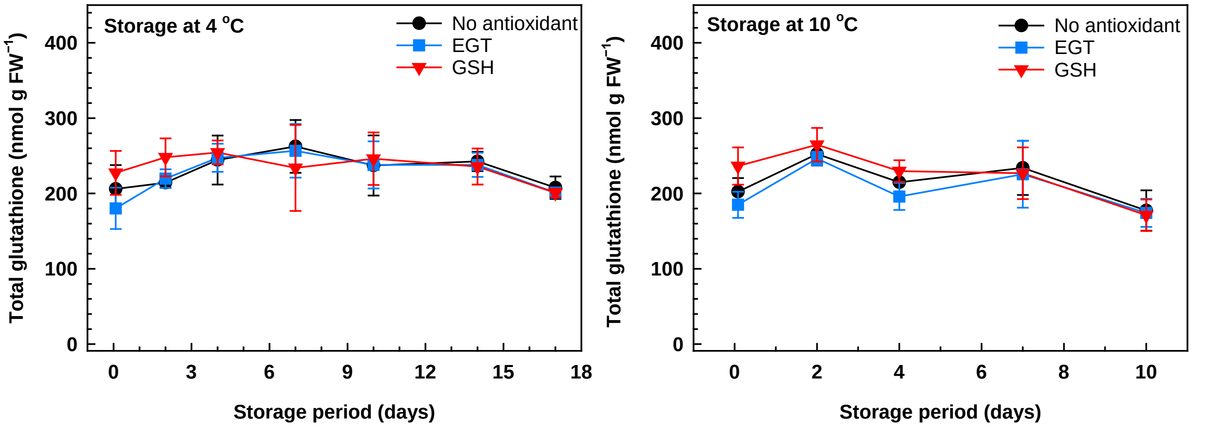

Figure S6. Total glutathione concentrations in arugula immersed in a pre-storage dip containing either 100 μM ergothioneine, 500 μM glutathione, or no antioxidant, and then stored at 4 °C or 10 °C.

All data are expressed on a fresh weight basis. Each datum represents the mean total glutathione concentration ± SE of three experimental replicates. Both plots are devoid of statistical lettering as there were no significant differences across treatments or their sampling days ($p \leq 0.05$).

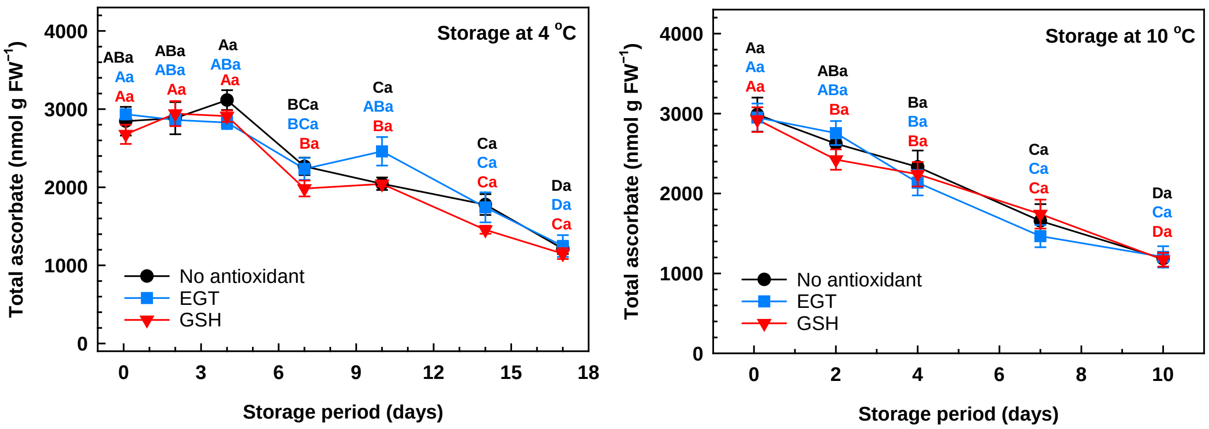

Figure S7. Total ascorbate concentrations in arugula immersed in a pre-storage dip containing either 100 µM ergothioneine, 500 µM glutathione, or no antioxidant, and then stored at 4 °C or 10 °C.

All data are expressed on a fresh weight basis. Each datum represents the mean total ascorbate concentration ± SE of three experimental replicates. Within each plot, uppercase letters denote statistical comparisons within a treatment across the storage period; lowercase letters denote statistical comparisons across the treatments at each postharvest sampling time. Shared letters represent no significant difference between means at $p \leq 0.05$.
